# Rapidly exploring structural and dynamic properties of signaling networks using PathwayOracle

**DOI:** 10.1186/1752-0509-2-76

**Published:** 2008-08-19

**Authors:** Derek Ruths, Luay Nakhleh, Prahlad T Ram

**Affiliations:** 1 Department of Computer Science, Rice University, Houston, Texas, USA; 2 Department of System Biology, University of Texas MD Anderson Cancer Center, Houston, Texas, USA

## Abstract

**Background:**

In systems biology the experimentalist is presented with a selection of software for analyzing dynamic properties of signaling networks. These tools either assume that the network is in steady-state or require highly parameterized models of the network of interest. For biologists interested in assessing how signal propagates through a network under specific conditions, the first class of methods does not provide sufficiently detailed results and the second class requires models which may not be easily and accurately constructed. A tool that is able to characterize the dynamics of a signaling network using an unparameterized model of the network would allow biologists to quickly obtain insights into a signaling network's behavior.

**Results:**

We introduce *PathwayOracle*, an integrated suite of software tools for computationally inferring and analyzing structural and dynamic properties of a signaling network. The feature which differentiates *PathwayOracle *from other tools is a method that can predict the response of a signaling network to various experimental conditions and stimuli using only the connectivity of the signaling network. Thus signaling models are relatively easy to build. The method allows for tracking signal flow in a network and comparison of signal flows under different experimental conditions. In addition, *PathwayOracle *includes tools for the enumeration and visualization of coherent and incoherent signaling paths between proteins, and for experimental analysis – loading and superimposing experimental data, such as microarray intensities, on the network model.

**Conclusion:**

*PathwayOracle *provides an integrated environment in which both structural and dynamic analysis of a signaling network can be quickly conducted and visualized along side experimental results. By using the signaling network connectivity, analyses and predictions can be performed quickly using relatively easily constructed signaling network models. The application has been developed in Python and is designed to be easily extensible by groups interested in adding new or extending existing features. *PathwayOracle *is freely available for download and use.

## Background

Reconstructing cellular signaling networks and understanding how they work are major endeavors in cell biology. The scale and complexity of these networks, however, render their analysis using experimental biology approaches alone very challenging. As a result, computational methods have been developed and combined with experimental biology approaches, producing powerful tools for the analysis of these networks. These tools aid biologists in interpreting existing experimental findings, evaluating hypotheses, enumerating possible biological behaviors, and, ultimately, in quickly designing experiments that maximize the amount of useful information gained. By assisting biologists in maximizing the amount of information obtained from their experiments through improved experimental design and more thorough analysis of results, computational tools increase the pace of scientific discovery.

Biological network analysis can generally be classified as either *structural *or *dynamic *[1]. Structural analysis provides insights into global properties of the network, among them decomposition of the network into functional modules (e.g., [2]), enumeration of signaling paths connecting arbitrary protein pairs (e.g., [3-5]), and the identification of key pathways that determine the behavior of the network (e.g., [2,6-10]). Dynamic methods, on the other hand, simulate the actual propagation of signals through a network by predicting the changes in the concentration of signaling proteins over time. These predictions will be of varying degrees of resolution and accuracy, depending largely on the accuracy and level of detail of the model from which they are produced.

The prevailing methods for dynamic analysis involve systems of ordinary differential equations (ODEs) [11,12]. These approaches require kinetic parameters for the individual biochemical reactions involved in the signaling process. This requirement often poses a significant hurdle for researchers as the numerical values of such parameters are difficult to obtain and may be the object of the researcher's project in the first place. In [13], we presented a novel signaling network simulation method which uses a non-parametric Petri net model of network to predict the signal flow under various experimental conditions. Our simulation method uses a novel technique to approximate the interaction speeds and predicts the qualitative behavior of the signaling network dynamics.

The advantage of our method over ODEs is the wide availability of connectivity-based models of signaling networks, and the relative speed with which they can be constructed. Numerous databases exist which catalog known signaling interactions (e.g., [14-16]). Thus, the existence and type (activating or inhibition) of an interaction can often be inferred directly from literature and/or these databases. This presents a stark contrast to the kinetic parameters required by ODEs, the numerical values for many of which must be determined experimentally for each experimental condition and cell line of interest [2].

In this paper, we present the software tool *PathwayOracle*, an integrated environment for connectivity-based structural and dynamic analysis of signaling networks, supporting

• visualization of signaling network connectivity;

• two versions of the simulation method described in [13] where

- the first allows prediction of signal flow through a given network for a specific experimental condition, and

- the second predicts the difference in signal flow through a given network induced by two different experimental conditions;

• enumeration of the paths connecting arbitrary pairs of nodes in the network; and

• visualization of experimental concentration data on the signaling network display.

In future releases we plan on expanding capabilities in all three areas of analysis – dynamic, structural, and experimental – with a focus on providing effective ways of integrating results from each together.

*PathwayOracle *has been designed in a modular fashion in order to facilitate extension of existing capabilities and the addition of new features.

Since PathwayOracle's most distinctive analytical capability involves the signaling Petri net simulator, a new dynamic analysis technique for signaling networks, we first provide an overview of the signaling Petri net modeling approach. Then in subsequent sections, we focus on PathwayOracle and explain the architecture and core concepts underlying the tool and then examine the individual features, how they can be used, and how they compare to existing tools.

### The Signaling Petri Net Simulator

**Figure 1:**
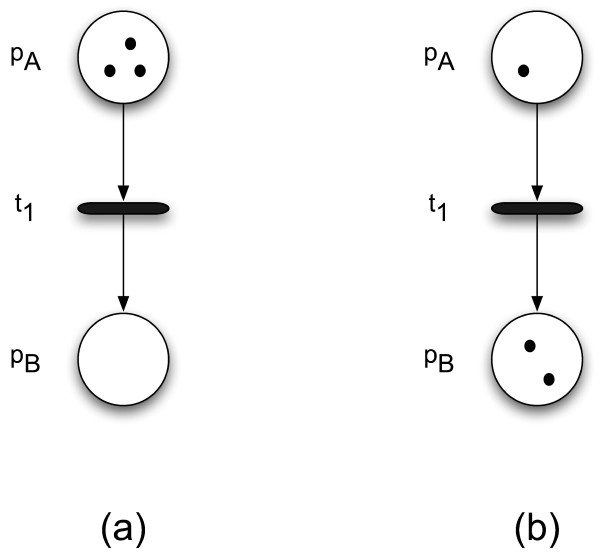
**An example of how tokens move among places**. In a Petri net, quantities of tokens are assigned to places. In (a), three tokens are assigned to place *p*_*A *_and zero tokens are assigned to place *p*_*B*_. The two places are connected by a transition, *t*_1_. The arcs in and out of *t*_1 _indicate the direction in which tokens move. When *t*_1 _fires, it moves some number of tokens from *p*_*A *_and puts them in *p*_*B*_. In (b), transition *t*_1 _has fired and moved two tokens from *p*_*A *_to *p*_*B*_.

Petri nets provide a graphical and executable model of processes in which information or material flows among a series of places or entities [17]. A Petri net consists of places, transitions, and tokens (see Figure 1). Quantities of tokens are assigned to individual places. This assignment is called a *marking*. As Figure 1 illustrates, the network flow is modeled by the reassignment of tokens to individual places in the Petri net in response to transition firings.

**Figure 2:**
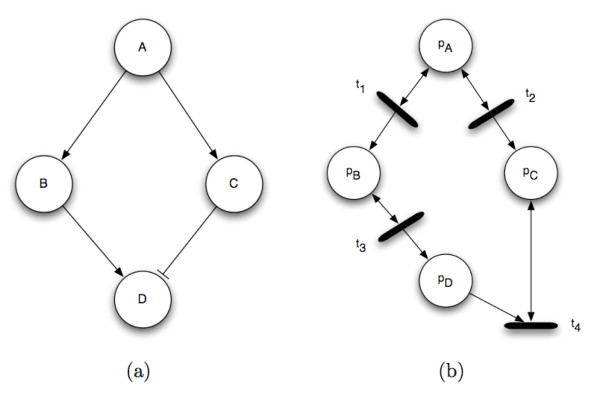
**An example signaling network and its corresponding Petri net**. An example signaling network (a) and its corresponding Petri net (b). Each signaling protein in the network, *A, B*, and *C*, is designated as a place *p*_*A*_, *p*_*B*_, and *p*_*C*_. A signaling interaction becomes a transition node and its input and output arcs. Note that the connectivity for an activating edge differs from that of an inhibitory edge.

A signaling Petri net is an extension of the Petri net formalism to model a signaling network. Places are signaling proteins and transitions implement directed protein interactions; each transition models the effect of a source protein on a target protein. The marking of (number of tokens in) protein *p *at time *t *is interpreted as the activity-level of that protein – the number of activated molecules of that type. Figure 2 shows the correspondence between a signaling network and a signaling Petri net model.

The signaling Petri net simulator models signal flow as the pattern of token accumulation and dissipation within proteins over time in the Petri net. Through transition firings, the source can influence the marking of (the number of tokens assigned to) the target, modeling the way that signals propagate through protein interactions in cellular signaling networks.

In order to overcome the issue of modeling reaction rates in the network, signaling dynamics are simulated by executing the signaling Petri net (SPN) for a set number of steps (called a *run*) multiple times, each time beginning at the same initial marking. For each run, the individual signaling rates are simulated via generation of random orders of transition firings (interaction occurrences). When the results of a large enough number of runs are averaged together, we find that the change in distribution of tokens in the network correlate with experimentally measured changes in the activity-levels of individual proteins in the underlying signaling network. In essence, the tokenized activity-levels computed by our method should be taken as abstract quantities whose changes over time correlate to changes that occur in the amounts of active proteins present in the cell. It is worth noting that some of the most widely used experimental techniques for protein quantification – western blots and microarrays – also yield results that are treated as indications, but not exact measurements, of protein activity-levels within the cell. Thus in some respects, the predictions returned by our SPN-based simulator can be interpreted like the results of a western blot or microarray experiment looking at changes relative to "control".

During a simulation run, the simulator imposes a strict ordering on transition firing such that it creates a two-time scale simulation. The smaller time scale is discretized as the firing of a single transition. This unit is referred to as the *firing *time scale. Firing steps are nested within a larger time scale, called time *blocks*, within which each transition is fired exactly once. The values returned by the simulator are the averaged token-counts for each protein at each time-block (across all runs).

**Figure 3:**
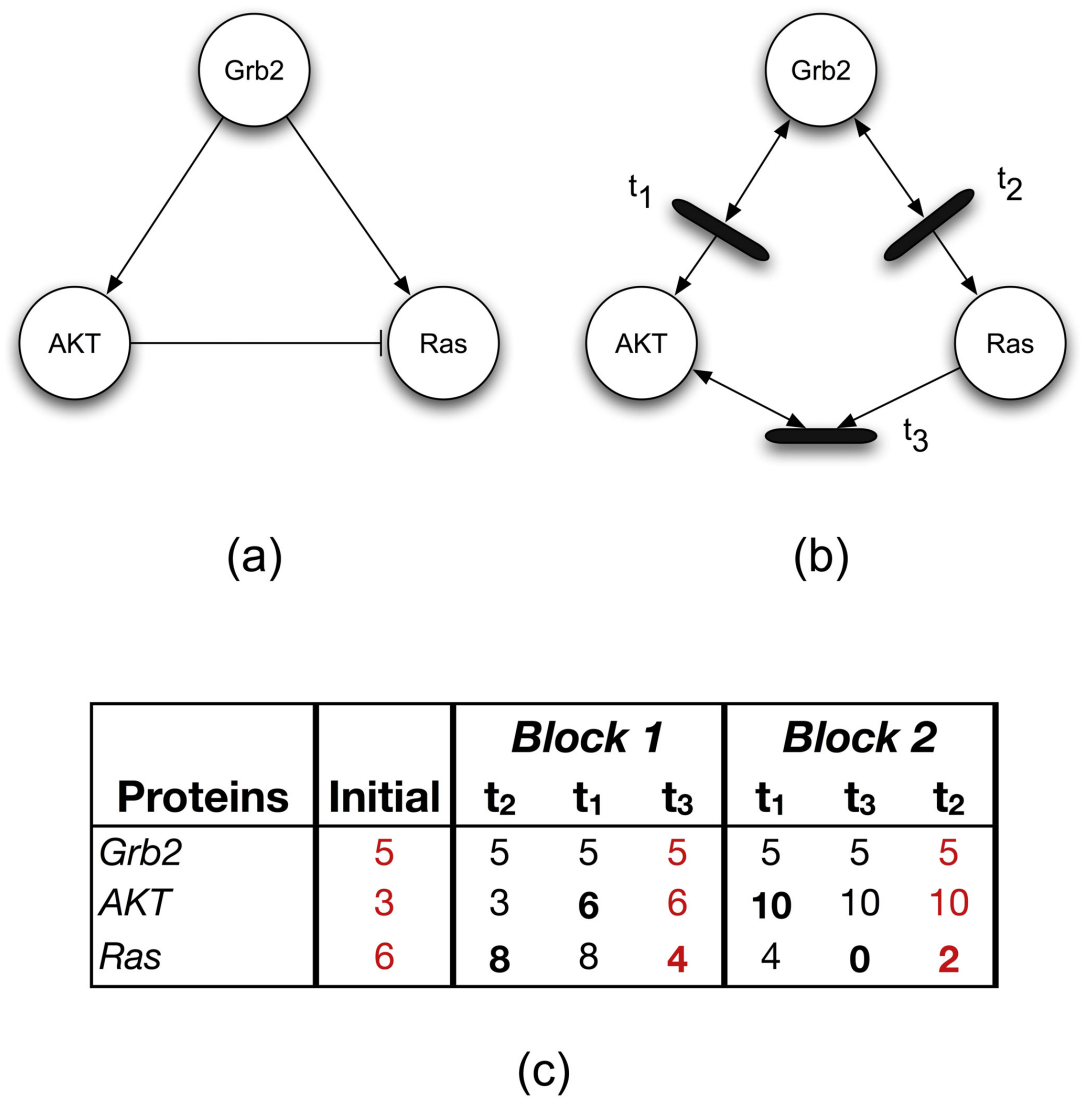
**An example signaling Petri net simulation**. (a) is the signaling network being simulated. (b) is the signaling Petri net that models that signaling Petri net. The table in (c) provides the markings for the Petri net over the course of a simulation run whose duration is two time blocks. The proteins are given the initial marking shown in the *Initial *column. Each subsequent column corresponds to a single time step during which one transition fired, producing a new marking of the network. The bold number in each column indicates which protein's marking was affected by the transition that fired in that time step. The red columns – always the last time step in the block – highlight the markings whose values would be averaged and used as part of the final result. These red columns are the sources of the markings that *PathwayOracle *reports.

Figure 3 provides a small example of a simulation run whose duration is two time blocks. As mentioned previously, within a given time block, each transition fires exactly once. Thus, in the table (Figure 3(c)), there is one column for each transition in each time block. The ordering of the transitions is shuffled in each time block in order to sample a different set of signaling rates within the networks.

In the first time block, transition *t*_2 _fires first: it reads 2 tokens out of *Grb2 *and places 2 additional tokens in *Ras*. Transition *t*_1 _fires second, reading 3 tokens out of *Grb2*. Transition *t*_3 _is evaluated last. The final marking for the network, highlighted as the red column in block 1 is used by the simulator as the marking for that block when averaging across runs.

At the conclusion of block 2, compare the values highlighted in red in the Initial column and at the end of both blocks. Note how the distribution of tokens have changed over the course of the simulation. *Grb2 *has the same number of tokens, implying that its activity-level has remained unchanged – this is consistent with the signaling network since no activating or inhibiting edges affect it in the model. *AKT*s token-count has risen, consistent with the fact that it is only activated in the signaling network. *Ras*'s token-count has fallen which is one plausible behavior of the system since it is activated by *Grb2*, but inhibited by *AKT*.

## Implementation

*PathwayOracle *is written in Python [18]. The user experience is oriented around visualization of and interaction with three main types of data: the signaling network, markings, and paths. At any given time, one signaling network is open, which is the basis for all analyses. Any simulation or concentration data is loaded and inspected as markings. Currently all static analyses revolve around paths, which are the third data type. In the following subsections, these individual data types and the user interfaces to them are discussed in more detail.

### The Signaling Network Model

While the implementation of our methods use the signaling Petri net model discussed in an earlier section of this paper, we provide a simpler and more convenient representation of the network to the user which omits the internal topology of the transitions and allows the user to specify interactions simply as either activating or inhibiting. Thus, for the remainder of this paper we use the following definition of the signaling network which is consistent with the experience the user will have when working with *PathwayOracle*. The signaling network connectivity is a directed graph *G *= (*V*, *E*) where

• *V *is the set of nodes, which are signaling proteins and complexes (hereafter referred to collectively as *signaling nodes*) and

• *E *is the set of edges, which are signaling interactions. Each edge is of one of two types: *u *→ *v *for activation and *u *⊣ *v *for inhibition.

Within *PathwayOracle*, each signaling node has a name, unique within the network. A signaling edge has no properties besides its type and is only defined by its *source *and *target*.

**Figure 4:**
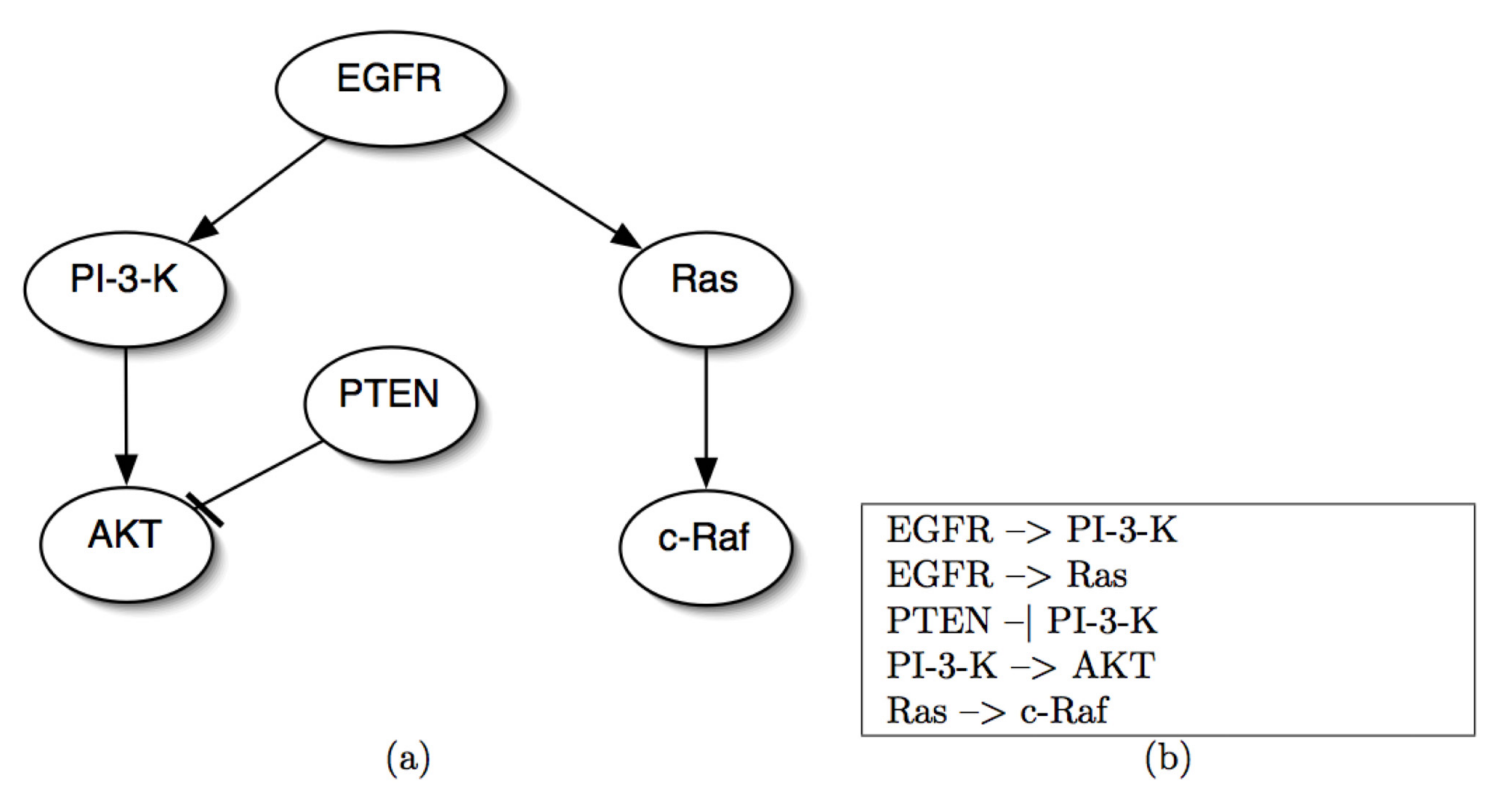
**An example of a Network in the Connectivity Format**. (a) A graphical representation of a signaling network's connectivity. (b) The signaling network in (a) written in the *Network Connectivity Format*.

In order to facilitate the rapid construction of such signaling network models, we devised a file format called the *Connectivity Format*. It is capable of expressing both general networks as well as paths. When representing a network in the format, as shown in the example in Figure 4(b), one signaling interaction is written on a line with the format

*u *-> *v*   or   *u *- | *v*

where *u *is the name of the source signaling node and *v *is the name of the target signaling node. Each node is taken to represent the active form of the protein it is named for. Thus, from the example above, the interaction PI-3-K→AKT means that the active form of PI-3-K increases the activity-level of AKT whereas the interaction PTEN⊣AKT means that the active form of PTEN decreases the activity-level of AKT. While these types of unparameterized relationships can be represented in SBML, SBML was designed for encoding much more information than just connectivity [19]. As a result, we deemed it appropriate to design a more concise format for our purposes. However, in a future release, *PathwayOracle *will support loading and saving in the SBML format.

At a given point in time, only one signaling network can be open in *PathwayOracle*. The main window displays a graphical representation of the network. The layout of the network can be modified by dragging nodes or by *shift*-clicking on edges to create, remove, or move waypoints. These layouts can be saved with the network and loaded again.

### Signaling Network Markings

In signaling networks, signal flow is measured and quantified as the fluctuation of concentrations of various forms of signaling proteins over time. In *PathwayOracle*, we model concentrations using the concept of a network *marking*, which was adapted from Petri nets in which it was first used [9].

#### Markings

In *PathwayOracle*, a marking, *μ *is an assignment of real values to the nodes of a signaling network such that every signaling node receives a value. Earlier, the concept of a marking was introduced as the assignment of tokens to protein places in the signaling Petri net. In a signaling Petri net, tokens are discrete. In *PathwayOracle*, a marking is an average of the markings from many independent simulation runs, which gives rise to the real, rather than integral values, assigned by the marking.

As discussed earlier, the value of the marking of a signaling node, *μ*(*v*), can be interpreted as an estimate of the concentration or change in concentration of the active form of the signaling protein *v *(we call the amount of the active form of the signaling protein its *activity-level*). The two different versions of the simulator generate markings with these different meanings. The first simulator predicts the signal flow due to an experimental condition and generates markings whose values are taken to represent the actual activity-level of signaling protein present over the assumed basal levels. The second version of the simulator predicts the difference in signaling due to changing experimental conditions. The values assigned by markings produced by this simulator correspond to the *change *in the activity-level of the protein induced by the change in experimental condition. This will be discussed further in the Results and Discussion section.

#### Marking Series

In order to model signal *flow*, a single marking is not enough since it only provides a single snapshot of concentrations throughout the network. A *marking series *is an sequence of markings, (*μ*_1_, *μ*_2_,..., *μ*_*T*_) in which the marking *μ*_*t *_is a snapshot of the concentration distribution at time step *t*. Thus, it is possible to see how the activity-level of protein *v *changed by plotting the values *μ*_1_(*v*), *μ*_2_(*v*),..., *μ*_*T*_(*v*). *PathwayOracle *provides the ability to do this.

**Figure 5:**
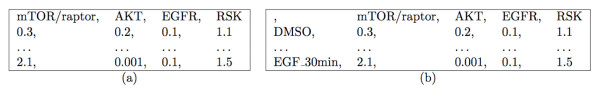
**Examples of marking series and group file formats**. (a) An example marking series dataset in the *comma-separated value *file format. The first row specifies the signaling proteins whose concentrations were measured. Each row thereafter specifies the concentration for a given time step: row *i *specifies the concentrations for each signaling protein at time step *i *- 1. (b) An example marking group dataset in the *comma-separated value *file format. The first row specifies the signaling proteins whose concentrations were measured. The first column specifies the names for each marking in the group dataset. The numbers in each row specify the concentration measured for each signaling protein in that marking.

*PathwayOracle *supports loading a marking series dataset from *comma-separated value *(*.csv*) files. As shown in Figure 5(a), the file has a header row which specifies, for each column, the name of the molecule whose concentration values will appear in that column. Each subsequent row contains the value assignments for a marking: the second row contains the marking for time step 1, the third row contains the marking for time step 2, and so on.

#### Marking Groups

In many experiments, the activity-level of various proteins are sampled at different time points and under different experimental conditions. Since the *marking series *is not able to represent changes due to different experimental conditions, we introduced the more general concept of a *marking group *in which each marking can correspond to an arbitrary activity-level distribution. Each marking is given a descriptive label that can be used to identify the conditions under which the activity-level was sampled.

Like the marking series, a marking group is loaded from a *.csv *file. However, unlike the marking series in which each row corresponds to a time step, in the marking group, each row corresponds to an independent marking (experimental condition). As shown in Figure 5(b), the first row is a header row specifying the molecule names for each column, the first column specifies the names for the individual markings (experimental conditions).

#### The Marking Manager

*PathwayOracle *includes a specific user-interface, the *Marking Manager*, designed to manage the three different types of markings. The Marking Manager provides a central interface within which it is possible to view all markings loaded and inspect them in ways that are relevant to their type (marking, marking series, or marking group). The specific ways in which markings can be inspected will be discussed further in the *Results *section.

### Signaling Paths

The current structural analysis capabilities available in *PathwayOracle *allow inspection of signaling paths within the network. A signaling path *p *is a sequence of nodes, (*v*_1_, *v*_2_,..., *v*_*k*_) where *v*_*i *_∈ *V *∀1 ≤ *i *≤ *k*, and (*v*_*i*_, *v*_*i *_+ 1) ∈ *E *∀1 ≤ *i *<*k*. In this case, we say that node *v*1 is the source of path *p*, and node *v*_*k *_is the *target *of *p*. Given a path, a variety of statistics may be of interest to the user. Additionally, it may be useful to view the path within the larger network. *PathwayOracle *provides these capabilites which will be discussed in the Results and Discussion section.

**Figure 6:**
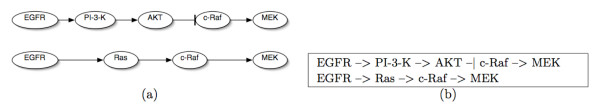
**An example of a Path in the Connectivity Format**. (a) A graphical representation of two signaling paths. (b) The signaling paths in (a) represented in the *Connectivity Format*. Each line corresponds to a single signaling path.

Sets of paths can be saved to a file and loaded back into a session. Like networks, paths are also stored in the Connectivity Format. When representing a set of paths, as shown in Figure 6, the full node names and the edge types are written so that all path information is directly available within the file itself. One line contains one path.

## Results

*PathwayOracle *provides a variety of tools for analyzing the structural and dynamic properties of a signaling network based on its connectivity. While its main differentiating feature is the ability to predict signal flow through a network using only the connectivity of the signaling network, *PathwayOracle *also provides the ability to visualize the network, analyze its connectivity, and inspect concentration-based experimental data.

**Figure 7:**
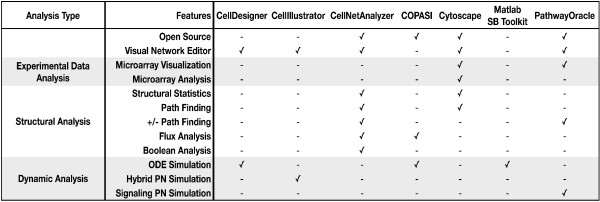
**A comparison of features supported by tools commonly used for signaling network analysis**. The table shows the features and analytical capabilities supported by different tools commonly used for the analysis of signaling networks. Tools included in the comparison are: CellDesigner [20], CellIllustrator [24], CellNetAnalyze [25], COPASI [22], Cytoscape [21], the System Biology Toolkit for Matlab [26], and PathwayOracle.

With the exception of the signaling Petri net simulator, PathwayOracle's features can be found in various combinations in other tools. Figure 7 provides a matrix of the features and capabilities of several tools most commonly-used for signaling network analysis. While other tools support a variety of simulation techniques, PathwayOracle, alone, provides non-parameterized simulation capabilities. It is worth noting that the commercial software package CellIllustrator [20] provides Petri net-based simulation capabilities. The difference between CellIllustrator and PathwayOracle Petri net approaches is the extensive set of kinetic parameters required by CellIllustrator in order to simulate a biological system. In this regard, hybrid functional Petri nets, the underlying technology used by CellIllustrator, are not significantly different from ODEs.

Another important distinguishing characteristic of PathwayOracle is the combination of features that it supports. Biological network analysis is a multi-faceted process that may involve structural, dynamic, and data analysis in parallel. Whereas other tools tend to focus on one or two of these general areas of analysis, we considered it important for PathwayOracle to incorporate all three in order to provide the researcher a single environment in which all their analysis could be done. In future releases we plan to increase PathwayOracle's support for all three of these directions of investigation: structural, dynamic, and data analysis.

In the remainder of this section, we discuss the features currently available in PathwayOracle.

### Network Visualization

As in many other computational analysis tools for signaling networks (e.g., [20,21]), an interactive graphical representation of the signaling network connectivity is at the center of the *PathwayOracle *interface. The main window provides a visualization of the signaling network connectivity. This visualization interface allows the user to edit the layout of the network by clicking on and dragging nodes and by *shift*-clicking on edges to create, remove, or move waypoints. Waypoints are points that lie on an edge. Holding down *shift *will display all edge waypoints. Existing waypoints can be dragged to change the path that an edge follows. Right-clicking on a waypoint will remove it. Left-clicking on a straight segment of the edge will create a new waypoint.

The network visualization also provides a view onto which path and experimental data analysis may be mapped. As will be discussed in subsequent sections, selected paths may be highlighted in this view and markings from experiments can set the colorings of individual nodes.

### Network Signal Flow Simulation

The main feature differentiating *PathwayOracle *from other tools, such as CellDesigner [20] and COPASI [22], is its ability to simulate signal flow using an unparameterized signaling network model. Simulations can be performed in two different ways. In the first (*Single Simulation*), the simulator predicts the signal flow through the network for a specific experimental condition. In the second (*Differential Simulation*), the simulator predicts the difference in signal flow due to two different experimental conditions on the same network. These simulation methods themselves are described in [13]. Here we focus on how simulations are configured, run, and analyzed.

Whereas the consensus networks typically represent the connectivity in normal cells, many experiments are conducted on abnormal cells in which oncogenic mutations, gene knockous, and pharmacological inhibitors have altered the behavior of various signaling nodes in the network. In *PathwayOracle *users can model these cell- and experiment-specific conditions by specifying each signaling node as either *High*, *Low*, or *Free*. The *High *state models any condition under which a protein's activity-level is held high for the duration of the experiment. This may be due to external stimulation or a known mutation in the protein that makes it constitutively active, for example. Similarly, a *Low *state models any phenomenon that forces a protein to have a persistently suppressed activity-level. This may be due to mutations that render the protein inactive, gene knockouts, or pharmacological inhibitors that force the activity-level of the protein low. In general, most signaling nodes will be *Free*, which means that their activity-level is unconstrained throughout the simulation. Only those nodes designated as *High *or *Low *will have their activity-level fixed for the duration of the simulation.

**Figure 8:**
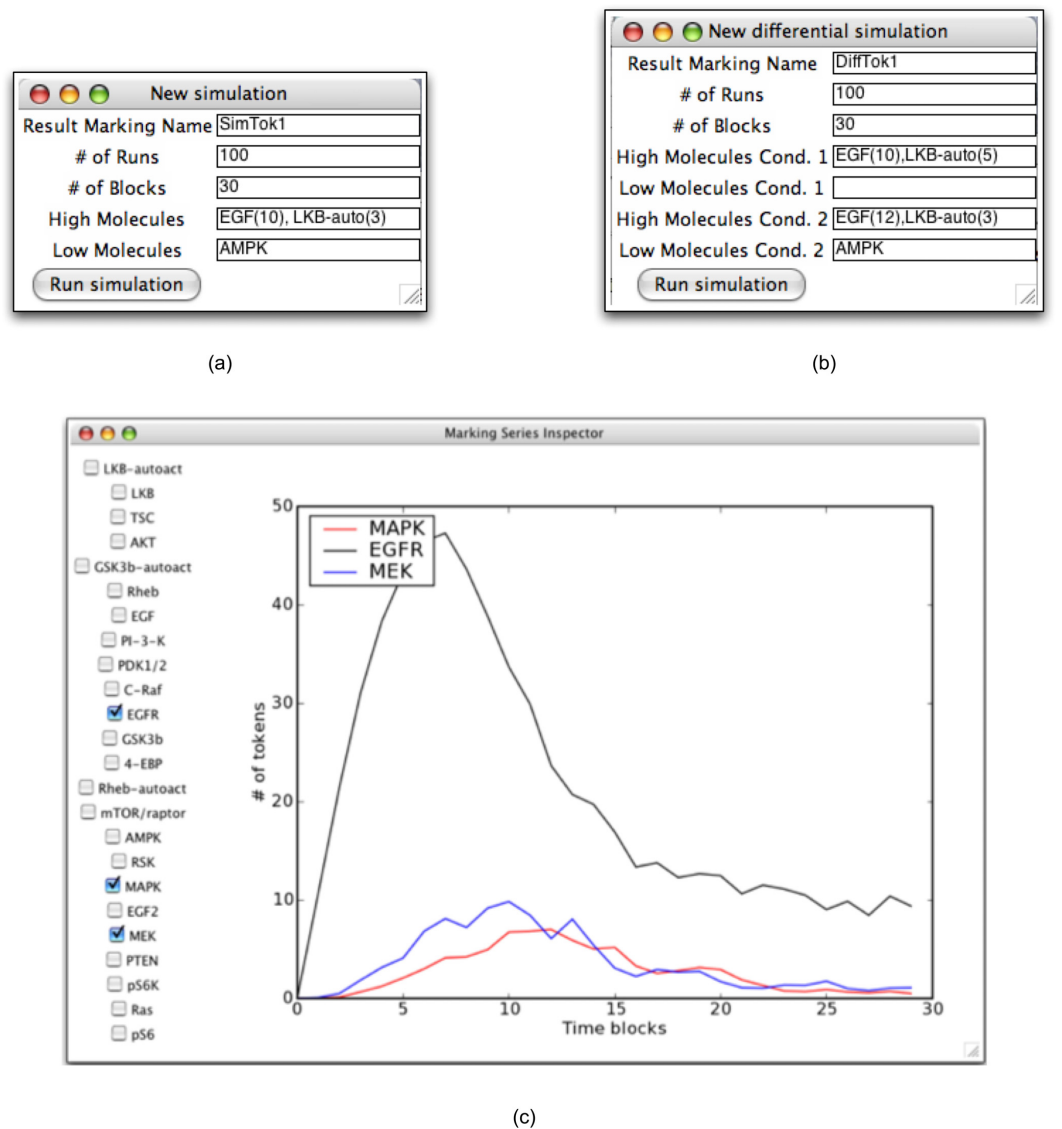
**The tokenized simulator user interface**. (a) The setup window for the tokenized simulator. The simulation is being configured to have two High nodes, EGF and LKB-auto. EGF will be initialized with a token-count of 10, LKB-auto with a token-count of 3. The token-count of AMPK will be zero for the duration of the simulation. (b) The setup window for the differential simulator. Two different scenarios are being compared through simulation: different token assignments are being tried with EGF and LKB-auto, with and without AMPK being fixed low. (c) The plot window for the marking series generated by a simulation. Observe that the signaling nodes whose activity-levels are plotted correspond to those selected in the checklist directly to the left of the plot.

In order for a protein to be held high during the simulation, it is necessary to indicate the initial activity-level that the protein will be elevated to. This is done by specifying the number of tokens that the protein will receive. Since a protein with a *High *state cannot be inhibited (even if inhibitory edges target it in the actual network), the protein's activity level will never fall below this initial value. The initial value for a High protein is indicated by placing it in parentheses next to the protein's name, as shown in Figure 8. Two other parameters that must be specified for a simulation are:

• the number of simulation runs to perform and

• the number of time blocks

The number of runs sets the number of independent simulations whose time block markings are averaged together to yield the overall simulation markings. In general, using more runs is a tradeoff between reliability of the results and simulation speed. In practice, the number of runs needed is dependent on the signaling network model and should be selected by observing the reproducability of the simulation results. An appropriate number of iterations will be large enough so that for a given experimental condition, the results are very similar across multiple simulations.

The time block, as discussed earlier, is a fundamental unit of time in the simulator. The appropriate number of time blocks for which to simulate will vary depending on the size of the signaling network and the scale of the network behavior of interest. Generally it should be selected by running simulations for a variety of time block values and determining which yields the most biologically reasonable activity-level changes for a known protein. While this is a manual process in the current version of *PathwayOracle*, we are investigating automated methods for estimating the number of time blocks by training against experimental time series data.

In *PathwayOracle*, the setup window for the *Single Simulation *(see Figure 8(a)) prompts the user for a single experimental condition. The setup window for the *Differential Simulation *(see Figure 8(b)) prompts the user for two experimental conditions. Both simulators produce a marking series. The tokenized simulation marking series corresponds to the activity-level time series predicted for the specified experimental condition. The differential simulation marking series corresponds to the change in activity-levels over time produced by switching from experimental condition 2 to experimental condition 1. The marking series produced by a simulation can be accessed through the Marking Manager. Choosing to *inspect *a marking series will present the user with a blank plot. By selecting signaling nodes, the plot is populated by the marking series values for individual nodes over time, as shown in Figure 8(c).

While this plot generation capability exists in many other dynamic simulation tools, the simplicity of the model used for simulation and the speed with which a simulation runs set *PathwayOracle *apart from other tools which require specification of the numerical values of kinetic parameters for each reaction in the network of interest (e.g., [20,22]). *PathwayOracle*, because of its novel approach, does not have such requirements. It is worth noting, however, where *PathwayOracle *provides approximations of signal flow, an ODE generates the actual concentration changes using extremely detailed and accurate models of the underlying biochemistry. The simulators in *PathwayOracle *provide an attractive, time- and resource-saving alternative this more exhaustively parameterized techniques. In particular, *PathwayOracle's *features will benefit researchers interested in quickly assessing characteristics of signal flow in their network.

For some networks, biologists will have partial knowledge of kinetic parameters or of other biological details which the signaling Petri net model does not, at present, consider. By integrating this knowledge into the simulator, it may be possible to improve the simulator's predictions. We identify this as a direction for future investigation. As the signaling Petri net simulator is extended, these new capabilities will be incorporated in future releases of *PathwayOracle*.

### Signaling Path Analysis

**Figure 9:**
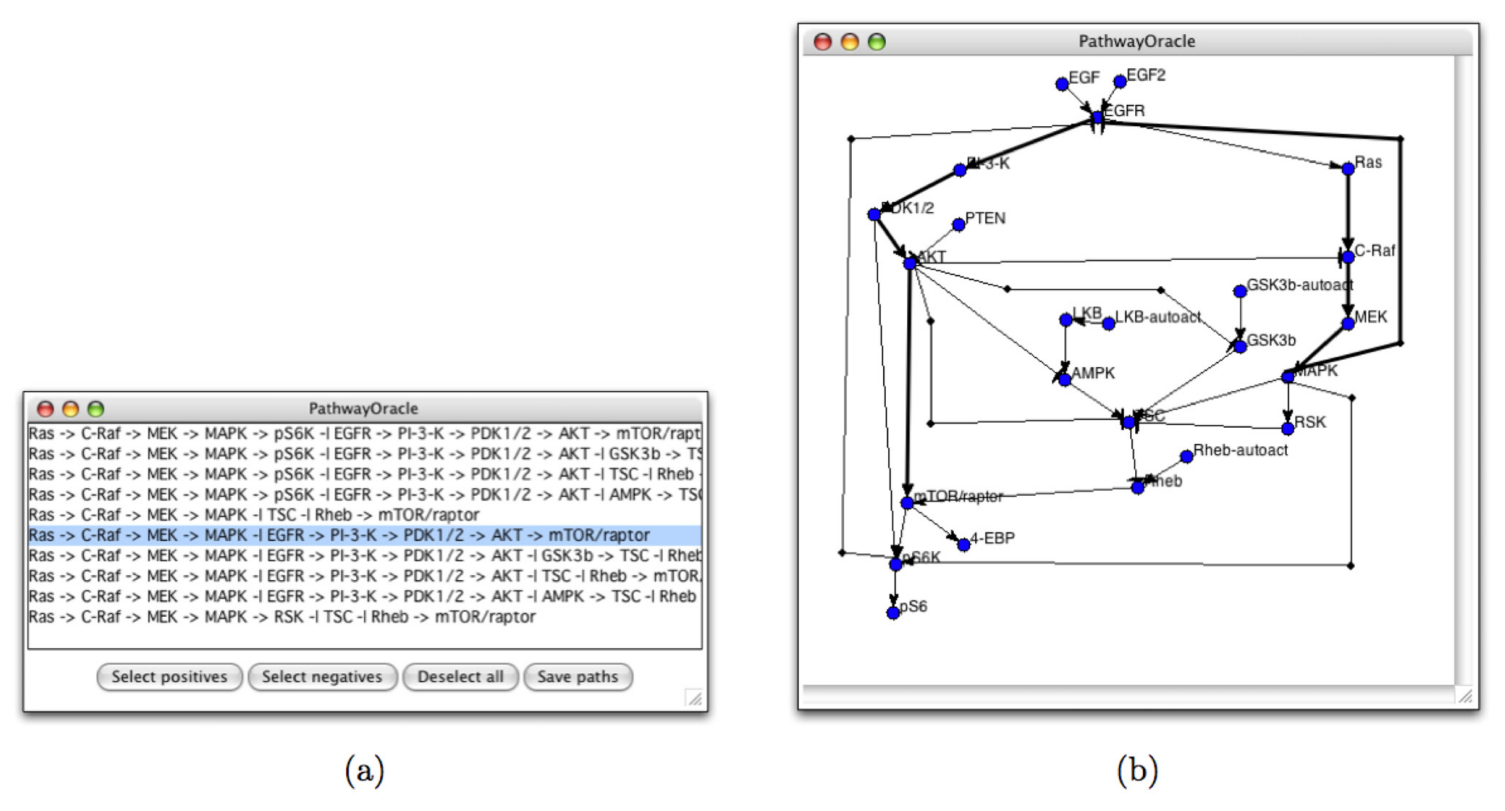
**The path interrogation user interface**. (a) The result window enumerating the set of all paths between *Ras *and *mTOR/raptor*. (b) The main network view showing the selected path highlighted.

The use of the simulators and plotting tools allows the user to observe trends in the activity-level of individual signaling nodes over time. Since the activity-level of a node is determined by the activity-level of other nodes in the network, the activity-level time series of a node may be explained by changes in the activity-level history of nodes upstream of it. In order to investigate such indirect interactions, it is useful to enumerate all the paths leading from a specific protein to the protein of interest. *PathwayOracle *provides this capability. Additionally, it provides various statistics on the set of paths linking two signaling nodes as well as a classification of the effect of each path as either *coherent *or *incoherent *(e.g. [23]). A coherent path is a directed series of interactions that leads from *x *to *y *such that an increase in the activity-level of *x *causes an increase in the activity of *y *and a decrease in the activity-level of *x *causes a decrease in the activity-level of *y*. An incoherent path is a directed series of interactions leading from *x *to *y *such that an increase in the activity-level of *x *causes a *d*ecrease in the activity-level of *y *and a decrease in the activity-level of *x *causes a *i*ncrease in the activity-level of *y*. It is possible to classify a path *p *as either coherent or incoherent by counting the number of inhibitory edges along *p*. *A path with an even number of inhibitory edges is coherent; a path with an odd number of inhibitory edges is incoherent *[5]. This logic is assumed in *PathwayOracle*. All simple paths (paths without loops) connecting two specified signaling nodes are enumerated by an exhaustive depth-first search. These paths then are classified as either coherent or incoherent, and presented to the user for further inspection in a window similar to the one shown in Figure 9(a). When a path is selected in the results window, it is highlighted in the main window, allowing the user to evaluate it within the context of the complete network (see Figure 9(b)).

### Experimental Data Analysis

**Figure 10:**
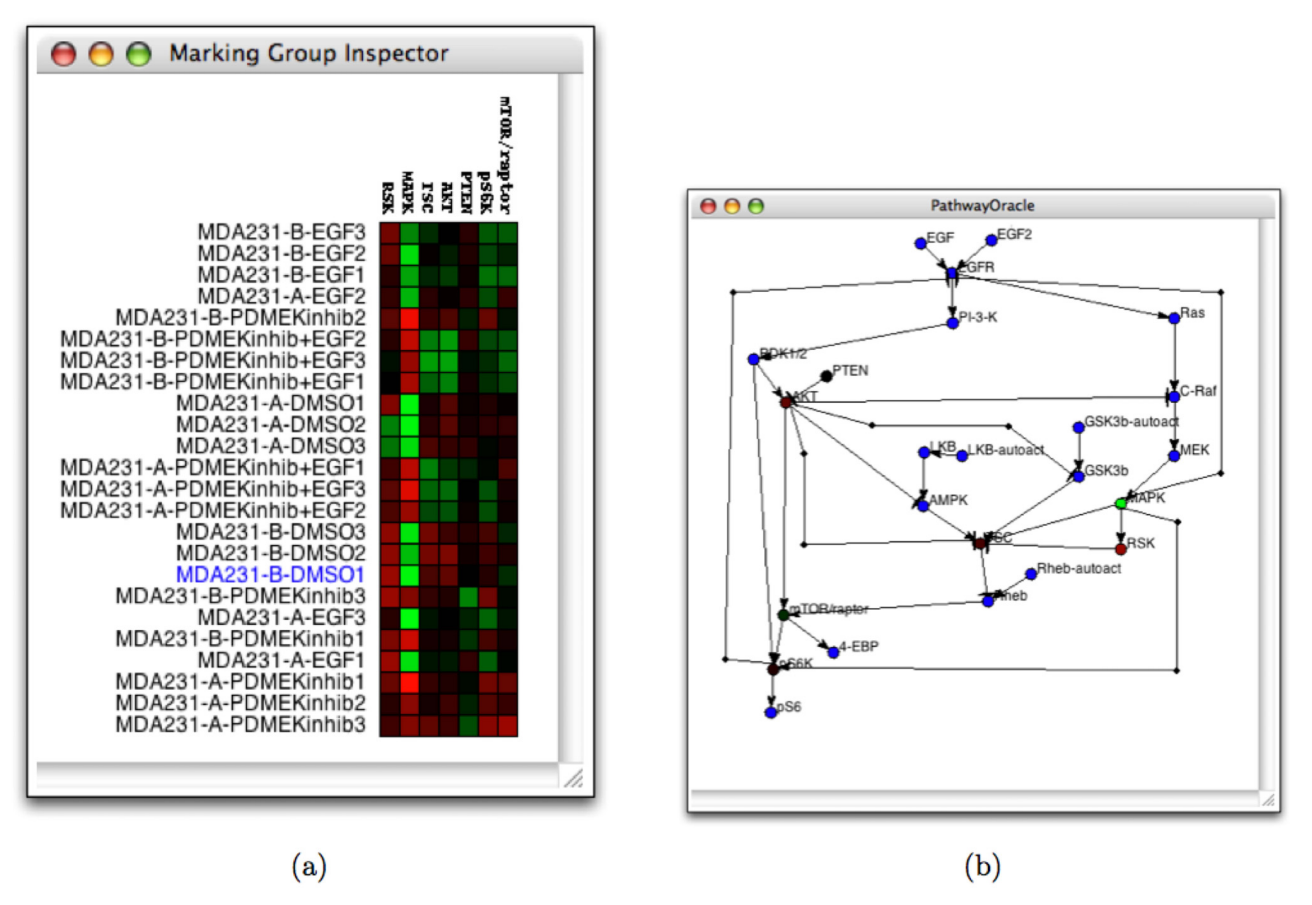
**The marking group user interface**. (a) The heat map visualization of a marking group. The selected marking, MDA231-B-DMSO1, is highlighted in blue. (b) The color distribution for the selected marking in the group is applied to the network view in the main window. Note that signaling nodes for which values were not given are not assigned a color on the valid red to green spectrum.

A model of the connectivity of a signaling network makes it possible to identify components of the model that are inconsistent with experimental data or visa versa. *PathwayOracle *enables this kind of analysis by allowing users to load experimental concentration data and visualize it both as a heatmap (see Figure 10(a)) or superimposed on the network view (see Figure 10(b)). Several other software tools provide similar capabilities (e.g., [21]). In *PathwayOracle*, experimental concentration data is loaded as a marking group in which a single marking corresponds to a condition for which concentrations were sampled. Figure 10(a) shows a marking group with 24 conditions (rows). The concentration of seven signaling proteins were sampled for each condition. This is the heatmap view for the marking group. When a specific marking in the group is selected, the colors for that marking are applied to the network view. This is particularly useful when assessing whether the experimental data is consistent with the interactions in the model. In Figure 10, the MDA231-B-DMSO2 marking has been superimposed on the network. We can see that RSK has a relatively low concentration despite the high concentration of MAPK. Given that, in the model, RSK is activated by MAPK, this combination of activity-levels seems unlikely to occur. Such an inconsistency suggests that there may be other signaling interactions contributing to the overall activity-level of RSK. Such an insight can help a researcher quickly identify areas where the model or experimental results need to be re-evaluated or improved.

### Future Directions

Our goal is to develop *PathwayOracle *into an integrated and expansive suite of tools that allow the biologist to extract as much information as possible from models of signaling network connectivity and experimental data relating to those models. We consider future directions for *PathwayOracle *to fall into several categories: network construction, network augmentation, experimental and computational analysis integration, and architecture.

One of the benefits of working with connectivity models of signaling networks is the abundance of databases and other online resources that publish connectivity-level data. Future versions of *PathwayOracle *will have support for querying such databases for connectivity components and, ultimately, for automated connectivity construction based on a set of signaling nodes specified by the user.

Analysis of network connectivity and topology is increasingly relevant to biological research. We intend to expand PathwayOracle's structural analysis features to include the ability to search for and identify motifs in the signaling networks.

Network connectivity can also be inferred from experimental data, which provides another direction for research and development. By using experimental results to identify inconsistencies between experimental results and the current network model, it may be possible for *PathwayOracle *to augment the network with new connectivity based on hints supplied by experimental results. At present only experimental concentration data is supported. However, as experiments produce more information beyond concentration profiles of signaling nodes, we plan to expand the experimental data that *PathwayOracle *can load, visualize, and use as part of network analyses.

Experimental results can also provide computational analysis methods information that can improve their final predictions or decompositions. Taking advantage of the additional, potentially obfuscated, information present in experimental results to improve the results returned by computational tools is a major goal for future versions of *PathwayOracle*.

A longer term direction for *PathwayOracle *is the integration of transcriptional and metabolic network analysis. In the biological systems of interest, the behavior of any one of these networks is dependent on the characteristics of the other two. As a result, developing a complete understanding of signaling, transcriptional regulation, or metabolism depends in part on integrating knowledge from the others. Finally, an ongoing priority in the design of *PathwayOracle *is its role as an open platform for the development and deployment of new analytical capabilities by other groups. Currently *PathwayOracle *employes a modular architecture that facilitates easy integration of new functionality. However, in future releases we plan to expose a plugin interface which will make it easier to developers and researchers to develop and deploy tools within PathwayOracle.

## Conclusion

*PathwayOracle *is an integrated software environment in which biologists may conduct structural and dynamic analysis of signaling networks of interest. *PathwayOracle *is distinguished from other tools in the field of systems biology by its ability to predict the signal flow through a network using a simplified, connectivity-based model of the signaling network. Simulations are fast and, based on a published study, predictors of signal propagation. This novel simulation capability, combined with support for structural analysis of connectivity between pairs of proteins and for analysis of certain kinds of experimental data make *PathwayOracle *a powerful asset in the experimentalist's endeavor to gain a more complete understanding of the cellular signaling network.

## Availability and requirements

**Project name: **PathwayOracle

**Project home page: **

**Operating system(s): **Platform independent

**Programming language: **Python

**Other requirements: **Python 2.4 or higher

**License: **GNU GPL

**Any restrictions to use by non-academics: **None

## Authors' contributions

DR designed and developed the PathwayOracle application, participated in evaluating features for inclusion, and drafted the manuscript. LN participated in application design and feature selection. PTR contributed biological case studies and data for PathwayOracle feature design. All authors read and approved the final manuscript.
